# Risk Groups and Psychosocial Factors for the Pandemic (COVID-19)

**DOI:** 10.3390/healthcare12131241

**Published:** 2024-06-21

**Authors:** Elif Yöyen, Ümmühan Deniz Sinanoğlu, Tülay Güneri Barış

**Affiliations:** 1 Department of Psychology, Faculty of Humanities and Social Sciences, Sakarya University, Sakarya 54050, Turkey; 2 Department of Clinical Psychology, Institute of Social Sciences, Maltepe University, Istanbul 34858, Turkey; 3 Department of Health Sciences, Institute of Business Administration, Sakarya University, Sakarya 54050, Turkey

**Keywords:** COVID-19, pandemic-related anxiety, risk groups for the pandemic, psychosocial factors

## Abstract

COVID-19, which started in 2019 and affected the whole world, has affected everyone at different intensities and in different ways. COVID-19, which is considered a pandemic, has turned into a major public health problem in terms of its consequences and has affected people biopsychosocially. However, people in risk groups may be affected more. This study was conducted to reveal the risk groups for the pandemic and to determine the psychosocial factors. Data were collected online using the relational screening model and snowball sampling methods. A Sociodemographic Information Form, COVID-19 Pandemic Psychosocial Impact Scale (C19-PPIS), and International Personality Inventory Short Form (IPISV) were sent online to 826 participants. Data were analysed using an independent sample *t*-test, a one-way ANOVA test, and the Pearson Correlation analysis. According to the results, young adults ($\bar{X}$ = 2.77), women ($\bar{X}$ = 2.79), singles ($\bar{X}$ = 2.78), those who are unemployed ($\bar{X}$ = 2.89), and those who had to change their home or city due to the pandemic ($\bar{X}$ = 2.89) were more affected by the pandemic. Psychological support was the support system needed the most during the pandemic ($\bar{X}$ = 3.04). In addition, a negative relationship was found between an extroverted personality and psychosocial impact from the pandemic (r = −0.148 and *p* < 0.01). A positive relationship was found between introversion (r = 0.183 and *p* < 0.01), agreeableness (r = 0.078 and *p* < 0.05), hostility (r = 0.094 and *p* < 0.01), disorganisation (r = 0.237 and *p* < 0.01), openness to development (r = 0.80 and *p* < 0.05), closed off to development (r = 0.070 and *p* < 0.05), emotional instability personality (r = 0.498 and *p* < 0.01), and psychosocial impact from the pandemic. This study has revealed important results regarding who has been most affected psychosocially by COVID-19. It is thought that the results obtained can guide state policies on what should be done in the field of preventive community mental health in another possible epidemic.

## 1. Introduction

COVID-19, which spread rapidly all over the world after emerging in December 2019, was declared as a pandemic by the World Health Organization (WHO) in 2020 [1]. The pandemic, which gave the whole world a hard time, negatively affected individuals and societies in many areas, especially health, economy, education, social well-being, and psychological well-being [2]. Studies conducted worldwide including Turkey, the country where this research was conducted, show that people have experienced depression, post-traumatic stress disorder, anxiety disorders, sleep problems, and burnout during the pandemic period [3,4,5,6,7]. In addition to psychopathology, it has also been reported that people experience death anxiety due to pandemic stress [8]. Stressful situations have a negative effect on thoughts and cause problems, especially in emotional regulation and interpersonal relationships [9]. Physical symptoms caused by traumatic stress and functional disorders can occur due to depression and anxiety caused by pandemic-related stress [10]. Since the pandemic is a social phenomenon affecting the individual and society as well as a health-related condition, it can be considered that a multidimensional psychosocial evaluation is needed in individuals affected by the pandemic, apart from the development of psychopathology alone.

Pandemics cause different psychosocial reactions because people experiencing panic and stress may exhibit different behaviours than normal, as the perception of the threat posed by the infectious disease increases. This state of panic and stress does not only belong to the individual, but may also become apparent to their relatives. The risk of contracting the disease, not knowing when the pandemic will disappear, not being economically stable, uncertainties about the virus and life, and concerns about how to protect oneself and one’s family can cause uncontrolled anxiety. In addition, when the social disadvantages brought by the pandemic are also involved, the situation may be more challenging for people. In the end, similar measures were taken to control the pandemic all over the world including Turkey, the country where this study was conducted. With long-lasting restrictions, regulations, and social isolation rules, daily life has changed in almost every aspect. Due to the quarantine and restrictions imposed to control the pandemic, most people had to work from home. They stayed away from their relatives and social support networks due to social isolation. Education has largely shifted to online education, with students studying online from home. The elderly and those with chronic diseases were banned from going out. Shopping malls, cafes, hairdressers and beauty centres, gyms, cinemas, and theatre halls were closed. Individual and social needs were met over the internet. Out-of-town and international travel were banned. Restrictions imposed on all areas of life such as social, artistic, sports, educational, cultural, and working life caused difficulties for human beings, who are biopsychosocial beings.

Everyone’s stress response to challenging events and situations is not the same. In these differences, individual characteristics and personality factors may be an important feature in the response to stress. Demographic characteristics [11,12] and personality traits [13,14] are among the individual factors that affect the way individuals are affected by the pandemic. When the individual characteristics that play a role in the pandemic are examined, being in the low-income group, being a woman, being a health worker [8], experiencing economic anxiety [15], having children [16], having a chronic disease [17], and being young [18] are among the factors identified for those being more affected. Apart from this, personality may also be a factor. The set of feeling, thinking, and behaviour patterns specific to the individual is called personality [19]. Although there are many different views and theories about personality traits, the Five Factor Personality Theory brings a holistic perspective to personality [20]. According to the Five Factor Personality Theory, personality has five dimensions universally and each dimension consists of two poles. In the first pole, extraversion, emotional balance, openness to development, responsibility, and compatibility are located. The opposite poles of these are introversion, emotional instability, closedness to development, difficulty, and hostility [21]. Each individual has all of these personality dimensions at different levels. Personality dimensions play an important role in individuals’ processes of making choices, interpreting, reacting, and being affected by events [22]. Therefore, we need to be aware of personality traits to understand the levels and ways individuals are affected by the pandemic. Although there are many studies on the COVID-19 pandemic and psychological health [23,24,25], these studies focus on shared experiences. However, individual differences (personality and sociodemographic characteristics) are extremely important in the assessment of critical events such as pandemics [26]. Some people may be extremely negatively affected by the pandemic, while others may be affected less and in different areas. Identifying these differences and re-exploring the factors that cause them will shed light on the studies that need to be carried out to reduce the effects of the pandemic.

How demographic and personality characteristics of individuals are affected psychosocially by the pandemic, and the relationship between personality and psychosocial impact levels have not been systematically revealed [27]. In the literature, there are studies mentioning the psychosocial effects of COVID-19 such as financial difficulties, disruptions in education, psychological burden, lifestyle changes, and family problems [28,29,30]. However, these studies generally focused on people living in a certain region or having certain occupational groups. Therefore, it may not be appropriate to generalise the results of these studies.

While the psychosocial effects of the pandemic are discussed, it is noteworthy that studies on psychosocial impact levels are missing in the literature. In the light of the above literature information, examining individual factors in investigating the impact of COVID-19 on psychological health is important both in understanding the effects of the ongoing pandemic and in determining health policies in the national and global management of pandemic processes that may occur in the future. In this context, the aim of this study is to determine the level of psychosocial impact of COVID-19 in terms of sociodemographic and personality characteristics of individuals, and to reach the qualities that can be included in risk groups from the determined characteristics. In line with this purpose, there are two main questions. Firstly, do individuals’ levels of psychosocial exposure to COVID-19 differ according to sociodemographic characteristics? The second question of the study is as follows: Do individuals’ levels of psychosocial exposure to COVID-19 differ according to their personality characteristics? Differences determined according to sociodemographic and personality characteristics may be instructive about which individuals are more sensitive to the psychosocial effects of the pandemic.

## 2. Materials and Methods

### 2.1. Study Design

This research was designed in a relational screening model. The relational screening model is a model that aims to determine the existence and degree of change between two or more variables. The relationship between variables may arise from mutual or partial dependence, or it may arise under the influence of a third variable.

The data were collected between March 2021 and June 2021. The answers given by the sample to the question of whether they have psychological illnesses are based on their personal statements. No psychiatric assessment or review of hospital records was performed. The inclusion criteria for the study were to be between the ages of 18 and 60, to be able to use a phone or computer for online access, not to have a physical or psychological disorder that prevents them from reading and answering the questions alone, and not to take medication. Groups other than the specified age groups, those with alcohol and substance abuse, or on antidepressants, anxiolytics, and other psychiatric drugs were excluded from this study.

Ethical approval for this study was obtained from Maltepe University Ethics Committee on 5 March 2021 with the approval number 2021/07-12. In this study, which was carried out with 842 participants aged 18 years and over living in different provinces of Turkey, data were collected from the general population by the snowball sampling method. The snowball sampling method was used because participants in risk groups are a relatively difficult and unknown group to reach (due to questions such as whether there is a death in the family due to COVID-19 or whether there is a COVID-19-positive individual in the family).

The snowball sampling technique is used in cases where it is difficult to reach the units that make up the universe or where information about the universe (size and depth of information, etc.) is missing [31]. This technique focuses on people and critical situations where rich data can be obtained, and reaches the universe by following these people and critical situations [32]. In order to conduct snowball sampling, the researchers selected the first and key participants in the population who they thought had the most information about the situation under study. The selection of these respondents is based on the information in the literature that first and key respondents are generally considered important by different individuals and groups [33]. The researchers reached new participants by asking the selected participants who else they could interview. Volunteers who agreed to participate in this study completed the research tools electronically after reading and signing the informed consent form. Data were collected online via Google Forms. The scales were made available online and sent to the participants via social media communication channels. The consent form included information about the purpose of the study, estimated duration, conditions of participation, and confidentiality principles. Participation was voluntary and no identity information was obtained from the participants. It took an average of 20 min to fill in the research data. In the analysis of the research data, the demographic forms and scales of 16 participants were incompletely completed, so they were not included in the analysis, and the analyses were made with the data of 826 participants.

### 2.2. Data Collection Tools

#### 2.2.1. Sociodemographic Information Form

Firstly, a literature review was conducted to investigate the characteristics of risk groups during the pandemic process [8,11,12,15,16,17,18]. Then, a Sociodemographic Information Form was developed by the researchers. The following questions were included in the form: age, gender, educational status, marital status, employment status, whether they had chronic diseases, whether they received psychological support before and during the pandemic, whether there was a change in their working life and income during the pandemic, and whether there was a person in their family who got sick or died due to the pandemic.

#### 2.2.2. COVID-19 Pandemic Psychosocial Impact Scale (C19-PPIS)

The COVID-19 Pandemic Psychosocial Impact Scale, developed by Yöyen and Sina-noğlu in 2021, is a 5-point Likert-type self-report scale consisting of 33 items and 5 sub-dimensions (close relationships, functionality, death anxiety, somatic symptoms, and anxious thoughts). It is accepted that as the score obtained from the scale increases, the level of individuals being affected by the pandemic also increases [34].

In this study, the Cronbach’s alpha value of the scale was determined as 0.95. The reliability coefficients for the sub-dimensions of the scale were determined as 0.92 for close relationships, 0.90 for functionality, 0.95 for death anxiety, 0.89 for somatic symptoms, and 0.91 for anxious thoughts.

#### 2.2.3. International Personality Inventory Short Version (IPISV)

The validity and reliability studies of the Turkish version of the International Personality Inventory Short Version (IPISV), which was developed by Goldberg in 1999 based on the Five Factor Personality Theory, were carried out by Yöyen in 2016. As a result of the validity and reliability studies, Cronbach’s alpha values of the sub-dimensions of the scale ranged between 0.64 and 0.75, while this ratio was found to be 0.82 for the whole scale. Each sub-dimension of the scale is scored separately. The scale, which consists of a total of 40 items and 9 sub-dimensions (extraversion–introversion, agreeableness–hostility, conscientiousness–difficulty, emotional instability, and openness to development–closedness to development), is a self-report scale structured as a 5-point Likert scale. At the opposite end of each dimension, there are dimensions defining opposite traits: introversion for extraversion, emotional instability for emotional balance, hostility for agreeableness, openness for closedness to development, and disorganisation for responsibility. Accordingly, the characteristics of the extraversion dimension are being talkative, brave, boisterous, boisterous, defiant, sociable, spontaneous, flamboyant, energetic, adventurous, friendly, outspoken, loud, noisy, ambitious, dominant, and social. The characteristics of the agreeableness dimension are being warm, kind, co-operative, unselfish, flexible, honest, polite, forgiving, helpful, content, loving, gentle, kind-hearted, sympathetic, secure, generous, considerate, and harmonious. The characteristics of the responsibility dimension were defined as regular, durable, careful, responsible, hardworking, effective, adequate, prudent, attentive, punctual, practical, excellent, economical, prudent, serious, economical, and reliable. The characteristics of the emotional balance dimension are defined as being non-emotional and non-jealous, relaxed, objective, calm, balanced temperament, good-natured, stable, contented, secure, dignified, non-demanding, docile, and peaceful. The characteristics of the openness to development dimension are described as being intelligent, alert, curious, creative, analytical, deep thinking, artistic, insightful, investigative, humorous, creative, undertaking complex thinking, knowledgeable, intellectual, broad interests, versatile, original, profound, and cultured. The opposite pole of all dimensions includes their opposite characteristics [35].

In this study, Cronbach’s alpha coefficients of the scale were determined as 0.72 for extroversion, 0.69 for introversion, 0.73 for agreeableness, 0.67 for hostility, 0.70 for responsibility, 0.67 for difficulty, 0.84 for emotional instability, 0.70 for openness to development, and 0.70 for closed off to development.

### 2.3. Statistical Analyses

SPSS 24.0 was used for data analysis in this study. Before analysing the data, missing values, extreme values, and normality were examined. As a result of the normal distribution of the data, hypothesis tests were statistically tested at a 95% confidence level. An independent sample *t*-test was used to examine the differences between the scores obtained from the scales according to the paired groups, and a one-way ANOVA test was used to determine the differences between more than two groups. The Pearson Correlation analysis was applied to determine the relationships between independent and dependent variables.

## 3. Results

When the distribution of the participants according to their demographic characteristics is analysed, it is seen that 52.1% (430 *n*) of the participants were between the ages of 18 and 35, and 47.9% (396 *n*) were 35 years old and over. It was determined that 67.9% of the participants were female (561 *n*) and 32.1% were male (265 *n*). When their educational status was evaluated, it was determined that 18.5% of the participants had a high school degree (153 *n*), 63.8% had a bachelor’s degree (527 *n*), and 17.7% had a master’s degree or above (146 *n*). In total, 56.3% were married (465 *n*), 43.7% were single (361 *n*), 66.2% were working (547 *n*), 17.1% were not working (141 *n*), 7.9% were students (65 *n*), and 8.8% were retired (73 *n*). It was determined that 22.4% of the participants lived with their family (185 *n*), 6.1% with their children (50 *n*), 30.8% with their partners and children (254 *n*), 24.1% with their partners (199 *n*), and 16.7% alone (138 *n*). In total, 18.4% of participants had a chronic disease (152 *n*) and 81.6% did not have a chronic disease (674 *n*). In total, 47.2% (390 *n*) of the participants had children and 52.8% (436 *n*) had no children. The results are shown in Table 1.

According to the results of the research, participants aged 18–35 ($\bar{X}$ = 2.77) were more affected by the pandemic than participants aged 35 and over ($\bar{X}$ = 2.55). The female participants’ level of being affected by the pandemic ($\bar{X}$ = 2.79) is higher than male participants ($\bar{X}$ = 2.39). In addition, the rate of female participants having anxious thoughts about the pandemic ($\bar{X}$ = 2.70) is higher and statistically significant (t = 5.230 and *p* < 0.05) compared to male participants ($\bar{X}$ = 2.31). Single participants ($\bar{X}$ = 2.78) were more affected by the pandemic than married participants ($\bar{X}$ = 2.57). The results are presented in Table 2.

As seen in Table 3, those who moved to another house due to the pandemic ($\bar{X}$ = 2.89) had higher levels of being affected by the pandemic than those who did not make any changes ($\bar{X}$ = 2.62), and participants whose income decreased during the pandemic ($\bar{X}$ = 2.82) had higher levels of being affected by the pandemic than participants whose income did not change ($\bar{X}$ = 2.56). In addition, an increase was found in the scores of close relationships (t = 3.635), functionality (t = 4.146), death anxiety (t = 2.867), somatic symptoms (t = 3.915), and anxious thoughts (t = 2.070), which are the sub-dimensions of the psychosocial impact of pandemic scale in participants whose income decreased during the pandemic.

The difference between the participants’ level of psychosocial impact from the COVID-19 pandemic and their previous psychological help was found to be statistically significant (t = 6.126 and *p* < 0.05). Those who received psychological help in the past ($\bar{X}$ = 2.94) were more affected by the pandemic than those who did not ($\bar{X}$ = 2.56). Close relationships and anxious thoughts scores were higher in participants who received psychological help in the past. Those who currently received psychological help ($\bar{X}$ = 3.04) were more affected by the pandemic than those who did not ($\bar{X}$ = 2.62). In addition, an increase was found in favour of the participants who were already receiving psychological help in all sub-dimensions of the pandemic psychosocial impact scale’s close relationships, functionality, death anxiety, and anxious thoughts. The values for these dimensions are as follows: for close relationships (t = 2.637 and *p* < 0.05); for functionality (t = 3.312 and *p* < 0.05); for death anxiety (t = 3.088 and *p* < 0.05), and for anxious thoughts (t = 2.914 and *p* < 0.05). The results are presented in Table 4.

There was no statistically significant differences (*p* > 0.05) in the participants’ level of psychosocial impact from the COVID-19 pandemic, according to whether they had a relative who was COVID-19 positive and lost his/her life due to COVID-19. The results are presented in Table 5.

According to the results, the levels of anxious thoughts of the participants were found to be statistically significant according to their educational status, and this significance is in favor of the participants with a higher education level (F = 5.475 and *p* = 0.004). The anxious thoughts levels of those with a master’s degree and above ($\bar{X}$ = 2.34) were lower than those with a high school ($\bar{X}$ = 2.57) and a bachelor’s degree ($\bar{X}$ = 2.65). The COVID-19 Pandemic Psychosocial Impact levels of the participants showed statistical significance according to their work status (F = 9.835 and *p* = 0.000). This significant difference is in favour of the working group. Accordingly, the COVID-19 pandemic impact levels of employees ($\bar{X}$ = 2.59) are lower than those who are unemployed ($\bar{X}$ = 2.89) and students ($\bar{X}$ = 2.99). The results are shown in Table 6.

Participants’ COVID-19 Pandemic Psychosocial Impact levels were found to be statistically significant according to their working life during the pandemic period (F = 7.187 and *p* = 0.000). Accordingly, those who continued to go to work as before the pandemic ($\bar{X}$ = 2.43) had lower levels of COVID-19 Pandemic Psychosocial Impact than those who worked from home for a while ($\bar{X}$ = 2.73), those who worked from home continuously ($\bar{X}$ = 2.68), those who were unemployed ($\bar{X}$ = 2.90), and those who did not work ($\bar{X}$ = 2.76). The level of being affected by close relationships of the unemployed ($\bar{X}$ = 2.82) is higher than those who worked from home for a while ($\bar{X}$ = 2.43), those who worked from home continuously ($\bar{X}$ = 2.54), and those who do not work ($\bar{X}$ = 2.45). The results are shown in Table 7.

When the findings in Table 8 are examined, it is seen that there is a statistically significant difference in the participants’ COVID-19 Pandemic Psychosocial Impact levels according to whom they live with (F = 5.287 and *p* = 0.000). Accordingly, individuals living with their families are more likely to be affected by the pandemic at the level of close relationships ($\bar{X}$ = 2.88) than others including those living with their children ($\bar{X}$ = 2.53), those living with their partner and children ($\bar{X}$ = 2.57), and those living alone ($\bar{X}$ = 2.71). The level of close relationship of those living with their partner and children ($\bar{X}$ = 2.41) is lower than those living with their family ($\bar{X}$ = 2.66) and only with their spouse ($\bar{X}$ = 2.68). The level of functionality of those living alone ($\bar{X}$ = 2.98) is lower than those living with their family ($\bar{X}$ = 3.39) and children ($\bar{X}$ = 3.34). The death anxiety level of those living alone ($\bar{X}$ = 2.50) is lower than those living with their family ($\bar{X}$ = 2.87) and partner ($\bar{X}$ = 2.83).

According to the results of this research, the level of the COVID-19 Pandemic Psychosocial Impact on those who need psychological support ($\bar{X}$ = 3.00) is higher than those who need only economic support ($\bar{X}$ = 2.64), those who need only social support ($\bar{X}$ = 2.49), and those who need health support ($\bar{X}$ = 2.33). In addition, the levels of anxious thoughts of those who need psychological support ($\bar{X}$ = 2.83) are higher than those who need economic support ($\bar{X}$ = 2.48) and those who need social support ($\bar{X}$ = 2.45); those who need health support ($\bar{X}$ = 2.47) was higher than that. The results are presented in Table 9.

When the personality trait dimensions were analysed, there was a negative correlation between the total score of the COVID-19 Pandemic Psychosocial Impact levels and extraversion (r = −0.148 and *p* < 0.01), and a low positive correlation between the introversion personality trait (r = 0.183 and *p* < 0.01), a low positive correlation between the personality trait of agreeableness (r = 0.078 and *p* < 0.05), a low positive correlation between the personality trait of hostility (r = 0.094 and *p* < 0.01), a low positive correlation between the personality trait of disorganised (r = 0.237 and *p* < 0.01), a positive correlation between the personality trait of emotional instability (r = 0.498 and *p* < 0.01), a low positive correlation between the personality trait of being open to development (r = 0.080 and *p* < 0.05), and a positive correlation between the personality trait of being closed off to development (r = 0.070 and *p* < 0.05). The results are presented in Table 10.

## 4. Discussion

### 4.1. Results Related to Demographic Characteristics

This study shows that young adults aged 18–35, women, singles, those who changed their homes/cities during the pandemic, those who received psychological help before the pandemic, those who were in the process of psychotherapy/psychiatric support during the pandemic, those who needed psychological help during the pandemic (compared to other types), those with low educational status, the unemployed, and those living alone were more affected by the pandemic. These groups can be considered as risk groups. The findings are consistent with the literature.

According to the studies conducted during the COVID-19 period, it was revealed that the level of stress, anxiety, and depression experienced by people was higher in younger participants and lower in older participants. It is seen that the group with the highest levels of stress, anxiety, and depression symptoms are those under the age of 25 [36,37]. The reason why young people are more affected by the pandemic may be that they stay at home due to social isolation, lack of support from close social relationships, and decreased visibility in the social context. The continuation of the formal education process on digital platforms and the transition of workplaces to online working may cause young people to have difficulty in managing their work and home lives together. The curfew during the pandemic period caused a withdrawal from social life. This is a source of stress.

In many studies conducted during the COVID-19 pandemic, it is observed that women are more negatively affected than men and this effect is especially associated with anxiety symptoms, anxiety levels, stress symptoms, and depression symptoms [38,39]. In the study conducted by Nkire, in which the anxiety, stress, and depression levels of the participants were evaluated during the COVID-19 pandemic, it was found that the scores of single people on stress and depression scales were higher than married people, in line with the findings of the current study [40]. Many studies in the literature also support these findings [41,42]. In addition, there is evidence in the literature that people with higher mobility rates had higer rates of psychologically affected by the pandemic. It has been reported that behavioural and emotional problems and depressive symptoms were more common in people who changed their place of residence compared to those who did not [43].

In a study, the recurrence rate of anxiety disorders was found to be 23.5%, and 32.7% of recurrent individuals were found to have anxiety disorders again [44]. It can be considered that the COVID-19 pandemic may have caused psychological problems from an individual’s past to re-emerge or caused them to experience a new psychological problem. Therefore, it should be known that people with psychological problems in the past will be in the risk group. In addition, it is possible to say that it is also compatible with the finding that the most affected group in both the general level of affectedness and the anxious thoughts sub-dimension, which is another result of the research, consists of participants who need psychological support. This finding is understandable when it is considered that the participants who need psychological support already have stress factors or traumatic experiences in their lives, or their coping skills are inadequate. Individuals’ needs for psychological support should guide government officials in determining health policies both during the ongoing pandemic process and in pandemics and/or other stressful situations (natural disasters, wars, etc.).

There are very few studies in the literature examining the effects of the pandemic with education level, and the results of one of them contradict the results of this study. According to the findings of Jin et al. (2021), individuals with different education levels were similarly affected by the pandemic [44]. The results of the working participants can be explained by the elimination of the stress caused by the economic anxiety experienced by the individuals. There are studies in the literature that have reached findings parallel to the current research findings. In the study conducted by Robillard et al. (2020), it was proved that the economic stress experienced during the pandemic period affected people in line with this finding. In a study conducted in Japan, it was found that people who did not work during the pandemic period showed more severe anxiety and depression symptoms [45]. It is thought that this situation can be explained in two different ways. Firstly, the continuation of people’s current organisation may have created a protective effect against the increase in stress levels. Secondly, it seems possible to think that people use going to work as an area where they can be busy and socialise and reduce their existing stress levels. According to the findings of the present study, the impact levels of those who do not work in the close relationship dimension were found to be higher than the others (those who work from home, those who work from home, and those who do not work). This finding seems to be in line with the findings in the literature that work loss during the pandemic period increases the problems experienced in interpersonal relationships [46]. The findings of the study also show that COVID-19 Pandemic Psychosocial Impact levels are positively associated with income loss. In addition, for all sub-dimensions, it was observed that the impact levels of those who experienced income loss were higher than those who did not experience income loss. This finding obtained in the sub-dimension of close relationships is in parallel with the studies in the literature. In a study examining intimate partner violence and economic stress, it was found that economic stress affected the frequency of sexual and psychological violence [47].

### 4.2. Results of Personality Characteristics

It was determined that as the extraversion and responsibility scores of individuals increased, their level of psychosocial impact from the COVID-19 pandemic decreased; as the scores of introversion, agreeableness, hostility, disorganisation, emotional instability, openness to development, and closed off to development increased, their level of psychosocial impact from the COVID-19 pandemic increased. According to the Five-Factor Theory of Personality, extroverts tend to feel better about themselves in general and use their positive focus as a way to cope with stress. In addition, individuals with high extraversion traits grow more in a more severe trauma [35]. Considering this information in the literature, it can be interpreted that the extraversion personality trait creates a protective effect against stress due to the COVID-19 pandemic and reduces the level of psychosocial impact. The research results show that the responsibility personality trait stands out as a protective factor against the COVID-19 pandemic. The pandemic process has brought restrictions, changes, and innovations in many individual and social areas (change in the way of working, curfews and travel restrictions, social distancing and housing use, etc.). These changes may have played a protective role against the psychosocial effects of the pandemic, considering the personality traits of individuals with high responsibility scores such as self-discipline, planning, and striving to achieve the goal.

Introverted individuals avoid facing emotional problems caused by stress [23]. The increase in stress level with the pandemic [48] may have inevitably increased the rate of these individuals being affected by the pandemic. Emotional instability is characterised by restlessness, irritability, and impatience, and consists of frequently experiencing emotional ups and downs [24]. Considering that anxiety, depression, anger, and vulnerability form the basis of emotional instability, the fact that the pandemic is associated with depression, anxiety, and high stress levels may explain its impact on individuals’ personality traits [23]. It is stated that individuals who are closed off to development are conservative, traditional, and fixed-minded. Individuals with high levels of disorganisation are reported to have low levels of responsibility, a lack of discipline, and a lack of sense of duty. The characteristics of the hostility dimension are social incompatibility, resentment, and social alienation. Agreeableness, on the other hand, is defined by characteristics such as benevolence, mildness, and respectfulness [22]. The COVID-19 process may have increased the level of individuals being affected by the pandemic because it forced mild-tempered people who like to be with people to stay away from people, forced individuals in the disorganisation dimension to take responsibility in their irresponsible nature, and forced those who are closed off to development to adapt to the newly changing world. All these features are combined with the stressful nature of the pandemic process.

This research has some limitations: First of all, this is a cross-sectional study and the results have limitations in generalisability. Another limitation is that the study data were collected online. Collecting face-to-face data in future studies may be important in the comparability of results. In addition, collecting data online has limited the collection of data from individuals who do not have internet access. Another limitation is that the data are based on the statements of the participants. Collecting psychological effects face-to-face with clinical tests and batteries can provide a more accurate evaluation of psychological effects.

## 5. Conclusions

The pandemic is a public health question affecting the whole world. However, the pandemic’s ethics are not limited to health alone. It can have economic and social impacts, and devastating individual and social impacts in the short and long term. In this context, recognizing the risk factors for ongoing and future pandemic processes can reduce these devastating effects. In this study, groups with risk factors were tried to be determined. Youngs, women, singles, unemployments, people who changed home or city due to the pandemic, and all personality structures except the extrovert and responsible personality structure were more affected by the pandemic.

## Figures and Tables

**Table 1:** Characteristics of the sample.

Demographic Characteristics	Group	*n*	Percentage %
Age	18–35 years	430	52.1
	35 years and older	396	47.9
Gender	Female	561	67.9
	Male	265	32.1
Educational status	High school graduate	153	18.5
	Bachelor’s degree	527	63.8
	Master’s degree and above	146	17.7
Marital status	Married	465	56.3
	Single	361	43.7
Having a child	Yes	390	47.2
	No	436	52.8
Working status	Working	547	66.2
	Not working	141	17.1
	Student	65	7.9
	Retired	73	8.8
People living together	With their family	185	22.4
	With their children	50	6.1
	With their partner and children	254	30.8
	With their partner	199	24.1
	Alone	138	16.7
Chronic disorder status	Yes	152	18.4
	No	674	81.6
	Total	826	100.0

**Table 2:** Independent samples *t*-test according to age, gender, and marital status.

Sub-Dimensions	Variable	Category	*n*	$\bar{X}$	t	df
**Close relationships**	Age	18–35 years	430	2.55	0.347	824
		35 years and older	396	2.52		
	Gender	Female	561	2.54	0.179	824
		Male	265	2.53		
	Marital status	Married	465	2.51	−0.781	824
		Single	361	2.57		
**Functionality**	Age	18–35 years	430	3.25	1.334	824
		35 years and older	396	3.15		
	Gender	Female	561	3.19	−0.315	824
		Male	265	3.22		
	Marital status	Married	465	3.15	−1.446	824
		Single	361	3.27		
**Death anxiety**	Age	18–35 years	430	2.75	0.291	824
		35 years and older	396	2.73		
	Gender	Female	561	2.74	−0.278	824
		Male	265	2.76		
	Marital status	Married	465	2.73	−0.294	824
		Single	361	2.76		
**Somatic symptoms**	Age	18–35 years	430	2.33	−0.859	824
		35 years and older	396	2.39		
	Gender	Female	561	2.36	−0.047	824
		Male	265	2.36		
	Marital status	Married	465	2.37	0.330	824
		Single	361	2.34		
**Anxious thoughts**	Age	18–35 years	430	2.54	−1.090	824
		35 years and older	396	2.62		
	Gender	Female	561	2.70	5.230 *	824
		Male	265	2.31		
	Marital status	Married	465	2.61	1.032	824
		Single	361	2.54		
**COVID-19 Pandemic Psychosocial Impact**	Age	18–35 years	430	2.77	4.023 *	824
		35 years and older	396	2.55		
	Gender	Female	561	2.79	6.925 *	824
		Male	265	2.39		
	Marital status	Married	465	2.57	−3.756 *	824
		Single	361	2.78		

* *p* < 0.05.

**Table 3:** Independent sample *t*-test results for city/home change during the pandemic and income change during the pandemic.

Sub-Dimensions	Variable	Category	*n*	$\bar{X}$	t	df	*p*
**Close relationships**	City/home change in the pandemic	Did not make any changes	705	2.55	0.508	824	0.612
		Moved to another city/home due to the pandemic	121	2.49			
	Income change status in the pandemic	Income decreased	326	3.635	3.635	824	0.000 *
		No change in income	500	2.43			
**Functionality**	City/home change in the pandemic	Did not make any changes	705	3.20	0.103	824	0.918
		Moved to another city/home due to the pandemic	121	3.19			
	Income change status in the pandemic	Income decreased	326	3.40	4.146	824	0.000 *
		No change in income	500	3.07			
**Death anxiety**	City/home change in the pandemic	Did not make any changes	705	2.75	0.646	824	0.518
		Moved to another city/home due to the pandemic	121	2.68			
	Income change status in the pandemic	Income decreased	326	2.89	2.867	824	0.004 *
		No change in income	500	2.65			
**Somatic symptoms**	City/home change in the pandemic	Did not make any changes	705	2.33	−1.818	824	0.069
		Moved to another city/home due to the pandemic	121	2.50			
	Income change status in the pandemic	Income decreased	326	2.52	3.915	824	0.000 *
		No change in income	500	2.25			
**Anxious thoughts**	City/home change in the pandemic	Did not make any changes	705	2.56	−1.469	824	0.142
		Moved to another city/home due to the pandemic	121	2.70			
	Income change status in the pandemic	Income decreased	326	2.67	2.070	824	0.039 *
		No change in income	500	2.52			
**COVID-19 Pandemic Psychosocial Impact**	City/home change in the pandemic	Did not make any changes	705	2.62	−3.371	824	0.001 *
		Moved to another city/home due to the pandemic	121	2.89			
	Income change status in the pandemic	Income decreased	326	2.82	4.404	824	0.000 *
		No change in income	500	2.56			

* *p* < 0.05.

**Table 4:** Independent samples *t*-test results according to the status of receiving psychological help in the past and the status of currently receiving psychological help.

Sub-Dimensions	Variable	Category	*n*	$\bar{X}$	t	df	*p*
**Close relationships**	Status of receiving psychological help in the past	Yes	229	2.66	2.016	824	0.044 *
		No	597	2.49			
	Current status of receiving psychological help	Yes	88	2.82	2.637	824	0.009 *
		No	738	2.50			
**Functionality**	Status of receiving psychological help in the past	Yes	229	3.30	1.659	824	0.097
		No	597	3.16			
	Current status of receiving psychological help	Yes	88	3.57	3.312	824	0.001 *
		No	738	3.16			
**Death anxiety**	Status of receiving psychological help in the past	Yes	229	2.75	0.156	824	0.876
		No	597	2.74			
	Current status of receiving psychological help	Yes	88	3.10	3.088	824	0.002 *
		No	738	2.70			
**Somatic symptoms**	Status of receiving psychological help in the past	Yes	229	2.45	1.833	824	0.067
		No	597	2.32			
	Current status of receiving psychological help	Yes	88	2.52	1.699	824	0.090
		No	738	2.34			
**Anxious thoughts**	Status of receiving psychological help in the past	Yes	229	2.73	2.764	824	0.006 *
		No	597	2.52			
	Current status of receiving psychological help	Yes	88	2.87	2.914	824	0.004 *
		No	738	2.54			
**COVID-19 Pandemic Psychosocial Impact**	Status of receiving psychological help in the past	Yes	229	2.94	6.126	824	0.000 *
		No	597	2.56			
	Current status of receiving psychological help	Yes	88	3.04	4.550	824	0.000 *
		No	738	2.62			

* *p* < 0.05.

**Table 5:** Independent sample *t*-test according to whether one of the family members is positive for COVID-19 and whether the relative dies due to COVID-19.

Sub-Dimensions	Variable	Category	*n*	$\bar{X}$	t	df	*p*
**Close relationships**	Having a family member positive for COVID-19	Yes	314	2.47	−1.532	824	0.126
		No	512	2.58			
	Having a relative who died due to COVID-19	Yes	208	2.56	0.360	824	0.719
		No	618	2.53			
**Functionality**	Having a family member positive for COVID-19	Yes	314	3.22	0.340	824	0.734
		No	512	3.19			
	Having a relative who died due to COVID-19	Yes	208	3.23	0.424	824	0.671
		No	618	3.19			
**Death anxiety**	Having a family member positive for COVID-19	Yes	314	2.72	−0.469	824	0.639
		No	512	2.76			
	Having a relative who died due to COVID-19	Yes	208	2.75	0.168	824	0.866
		No	618	2.74			
**Somatic symptoms**	Having a family member positive for COVID-19	Yes	314	2.35	−0.042	824	0.967
		No	512	2.36			
	Having a relative who died due to COVID-19	Yes	208	2.46	1.748	824	0.081
		No	618	2.32			
**Anxious thoughts**	Having a family member positive for COVID-19	Yes	314	2.58	0.060	824	0.952
		No	512	2.58			
	Having a relative who died due to COVID-19	Yes	208	2.54	−0.705	824	0.481
		No	618	2.59			
**COVID-19 Pandemic Psychosocial Impact**	Having a family member positive for COVID-19	Yes	314	2.66	−0.029	824	0.977
		No	512	2.66			
	Having a relative who died due to COVID-19	Yes	208	2.61	−1.136	824	0.256
		No	618	2.68			

**Table 6:** ANOVA test results determining the change according to education status and working status.

Sub-Dimensions	Variable	Category	*n*	$\bar{X}$	F	*p*	Scheffe
**Close relationships**	**Educational status**	High school graduate	153	2.48	0.339	0.713	
		Bachelor’s degree	527	2.54			
		Master’s and above	146	2.58			
	**Working status**	Working	547	2.55	0.184	0.907	
		Not working	141	2.51			
		Student	65	2.47			
		Retired	73	2.59			
**Functionality**	**Educational status**	High school graduate	153	3.18	0.572	0.565	
		Bachelor’s degree	527	3.23			
		Master’s and above	146	3.12			
	**Working Status**	Working	547	3.22	0.834	0.475	
		Not working	141	3.24			
		Student	65	3.22			
		Retired	73	3.01			
**Death anxiety**	**Educational Status**	High school graduate	153	2.69	0.169	0.844	
		Bachelor’s degree	527	2.75			
		Master’s and above	146	2.76			
	**Working Status**	Working	547	2.76	0.702	0.551	
		Not working	141	2.81			
		Student	65	2.59			
		Retired	73	2.65			
**Somatic symptoms**	**Educational status**	High school graduate	153	2.44	1.336	0.264	
		Bachelor’s degree	527	2.36			
		Master’s and above	146	2.26			
	**Working status**	Working	547	2.35	0.838	0.473	
		Not working	141	2.45			
		Student	65	2.24			
		Retired	73	2.32			
**Anxious thoughts**	**Educational status**	High school graduate ^(1)^	153	2.57	5.475	0.004 *	(3-1) (3-2)
		Bachelor’s degree ^(2)^	527	2.65			
		Master’s and above ^(3)^	146	2.34			
	**Working status**	Working (1)	547	2.50	3.416	0.017 *	(1-2)
		Not working (2)	141	2.79			
		Student (3)	65	2.62			
		Retired (4)	73	2.70			
**COVID-19 Pandemic Psychosocial Impact**	**Educational status**	High school graduate	153	2.68	0.548	0.578	
		Bachelor’s degree	527	2.68			
		Master’s and above	146	2.60			
	**Working status**	Working (1)	547	2.59	9.835	0.000 *	(1-2) (1-3)
		Not working (2)	141	2.89			
		Student (3)	65	2.99			
		Retired (4)	73	2.47			

* *p* < 0.05. ^(1)^ High school graduate, ^(2)^ Bachelor’s degree, ^(3)^ Master’s and above for Educational status; (1) Working, (2) Not working, (3) Student, (4) Retired for Working status.

**Table 7:** ANOVA test determining the change according to working life.

Sub-Dimensions	Variable	Category	*n*	$\bar{X}$	F	*p*	Scheffe
**Close relationships**	**Working life in the pandemic**	Worked from home for a while ^(1)^	150	2.43	2.390	0.049 *	(4-1) (4-2) (4-5)
		Worked from home ^(2)^	189	2.54			
		Continued to go to work as before the pandemic ^(3)^	210	2.58			
		Was unemployed ^(4)^	87	2.82			
		Not working ^(5)^	190	2.45			
**Functionality**	**Working life in the pandemic**	Worked from home for a while ^(1)^	150	3.09	3.559	0.007 *	(4-1) (4-3) (4-5)
		Worked from home ^(2)^	189	3.33			
		Continued to go to work as before the pandemic ^(3)^	210	3.17			
		Was unemployed ^(4)^	87	3.51			
		Not working ^(5)^	190	3.07			
**Death anxiety**	**Working life in the pandemic**	Worked from home for a while	150	2.70	2.013	0.091	
		Worked from home	189	2.75			
		Continued to go to work as before the pandemic	210	2.69			
		Was unemployed	87	3.07			
		Not working	190	2.68			
**Somatic symptoms**	**Working life in the pandemic**	Worked from home for a while ^(1)^	150	2.19	2.380	0.050 *	(1-3) (1-4)
		Worked from home ^(2)^	189	2.33			
		Continued to go to work as before the pandemic ^(3)^	210	2.41			
		Was unemployed ^(4)^	87	2.55			
		Not working ^(5)^	190	2.36			
**Anxious thoughts**	**Working life in the pandemic**	Worked from home for a while ^(1)^	150	2.66	6.057	0.000 *	(3-1) (3-2) (3-4) (3-5)
		Worked from home ^(2)^	189	2.53			
		Continued to go to work as before the pandemic ^(3)^	210	2.32			
		Was unemployed ^(4)^	87	2.76			
		Not working ^(5)^	190	2.76			
**COVID-19 Pandemic Psychosocial Impact**	**Working life in the pandemic**	Worked from home for a while ^(1)^	150	2.73	7.187	0.000 *	(3-1) (3-2) (3-4) (3-5)
		Worked from home ^(2)^	189	2.68			
		Continued to go to work as before the pandemic ^(3)^	210	2.43			
		Was unemployed ^(4)^	87	2.90			
		Not working ^(5)^	190	2.76			

* *p* < 0.05^. (1)^ Worked from home for a while, ^(2)^ Worked from home, ^(3)^ Continued to go to work as before the pandemic, ^(4)^ Was unemployed, ^(5)^ Not working.

**Table 8:** ANOVA test regarding the variables with whom one lives.

Sub-Dimensions	Variable	Category	*n*	$\bar{X}$	F	*p*	Scheffe
**Close relationship**	**Cohabitant**	Family ^(1)^	185	2.66	2.922	0.020 *	(3-1) (3-4)
		Children ^(2)^	50	2.53			
		Partner and children ^(3)^	254	2.41			
		Partner ^(4)^	199	2.68			
		Alone ^(5)^	138	2.41			
**Functionality**	**Cohabitant**	Family ^(1)^	185	3.39	3.364	0.010 *	(5-1) (5-2)
		Children ^(2)^	50	3.34			
		Partner and children ^(3)^	254	3.14			
		Partner ^(4)^	199	3.23			
		Alone ^(5)^	138	2.98			
**Death anxiety**	**Cohabitant**	Family ^(1)^	185	2.87	2.486	0.042 *	(5-1) (5-4)
		Children ^(2)^	50	2.73			
		Partner and children ^(3)^	254	2.71			
		Partner ^(4)^	199	2.83			
		Alone ^(5)^	138	2.50			
**Somatic symptoms**	**Cohabitant**	Family	185	2.44	1.553	0.185	
		Children	50	2.47			
		Partner and children	254	2.35			
		Partner	199	2.38			
		Alone	138	2.19			
**Anxious thoughts**	**Cohabitant**	Family	185	2.62	0.506	0.732	
		Children	50	2.43			
		Partner and children	254	2.61			
		Partner	199	2.57			
		Alone	138	2.53			
**COVID-19 Pandemic Psychosocial Impact**	**Cohabitant**	Family ^(1)^	185	2.88	5.287	0.000 *	(1-2) (1-3) (1-4)
		Children ^(2)^	50	2.53			
		Partner and children ^(3)^	254	2.57			
		Partner ^(4)^	199	2.58			
		Alone ^(5)^	138	2.71			

* *p* < 0.05^. (1)^ Live with Family, ^(2)^ Live with children, ^(3)^ Live with partner and children, ^(4)^ Live with partner, ^(5)^ Live alone.

**Table 9:** ANOVA test regarding the variables type of support needed.

Sub-Dimensions	Variable	Category	*n*	$\bar{X}$	F	*p*	Scheffe
**Close relationship**	**Most-needed help in the pandemic**	Economic help	164	2.62	0.944	0.419	
		Psychological help	261	2.56			
		Social help	319	2.46			
		Health help	82	2.59			
**Functionality**	**Most-needed help in the pandemic**	Economic help	164	3.24	0.181	0.910	
		Psychological help	261	3.21			
		Social help	319	3.19			
		Health help	82	3.14			
**Death anxiety**	**Most-needed help in the pandemic**	Economic help	164	2.81	0.404	0.750	
		Psychological help	261	2.70			
		Social help	319	2.73			
		Health help	82	2.81			
**Somatic symptoms**	**Most-needed help in the pandemic**	Economic help	164	2.39	0.650	0.583	
		Psychological help	261	2.32			
		Social help	319	2.34			
		Health help	82	2.48			
**Anxious thoughts**	**Most-needed help in the pandemic**	Economic help ^(1)^	164	2.48	8.167	0.000 *	(2-1) (2-3) (2-4)
		Psychological help ^(2)^	261	2.83			
		Social help ^(3)^	319	2.45			
		Health help ^(4)^	82	2.47			
**COVID-19 Pandemic Psychosocial Impact**	**Most-needed help in the pandemic**	Economic help ^(1)^	164	2.64	26.227	0.000 *	(2-1) (2-3) (2-4)
		Psychological help ^(2)^	261	3.00			
		Social help ^(3)^	319	2.49			
		Health help ^(4)^	82	2.33			

* *p* < 0.05. ^(1)^ Most need economic help, ^(2)^ Most need psychological help, ^(3)^ Most need social help, ^(4)^ Most need health help in the pandemic.

**Table 10:** The Pearson Correlation analysis of the relationship between the COVID-19 Pandemic Psychosocial Impact Scale and Personality Inventory.

Scales	1	2	3	4	5	6	7	8	9
**COVID-19 Pandemic Psychosocial Impact**	−0.148 **	0.183 **	0.078 *	0.094 **	−0.019	0.237 **	0.498 **	0.080 *	0.070 *
**Close relationships**	−0.166 **	0.197 **	−0.024	0.127 **	−0.039	0.214 **	0.381 **	0.057	0.082 *
**Functionality**	−0.098 **	0.103 **	0.044	0.072 *	−0.045	0.193 **	0.419 **	0.075 *	0.039
**Death anxiety**	−0.104 **	0.082 *	0.120 **	0.038	0.045	0.135 **	0.382 **	0.091 **	−0.009
**Somatic symptoms**	−0.110 **	0.166 **	0.012	0.118 **	−0.077 *	0.245 **	0.428 **	0.054	0.106 **
**Anxious thoughts**	−0.092 **	0.171 **	0.122 **	0.027	0.004	0.160 **	0.337 **	0.024	0.078 *

** *p* < 0.01, * *p* < 0.05. Note, 1—extroversion; 2—introversion; 3—agreeableness; 4—hostility; 5—responsibility; 6—disorganised; 7—emotional stability/instability; 8—openness to development; and 9—closed off to development.

## Data Availability

The data presented in this study are available to all researchers.
